# Family matters: home characteristics related to dietary intake of fruit, vegetables and sugar-sweetened beverages in a national cross-sectional sample of school-aged children in the U.S.

**DOI:** 10.1186/s40795-026-01339-8

**Published:** 2026-05-02

**Authors:** Lorrene D. Ritchie, Alexander C. McLain, Marsha Dowda, Russell Pate, Edward A. Frongillo, Deborah Parra-Medina, Gail Woodward-Lopez

**Affiliations:** 1 Agriculture and Natural Resources, Nutrition Policy Institute, University of California, 1111 Franklin Street, Eleventh Floor, Oakland, CA 94607 USA; 2 Epidemiology and Biostatistics, Arnold School of Public Health, University of South Carolina, Discovery 456, 915 Greene Street, Columbia, SC 29208 USA; 3 Children’s Physical Activity Research Group, Exercise Science, Arnold School of Public Health, University of South Carolina, Public Health Research Center 251, 921 Assembly Street, Columbia, SC 29208 USA; 4 Health Promotion, Education and Behavior, Arnold School of Public Health, University of South Carolina, Discovery I, 915 Greene Street, Columbia, SC 29208 USA; 5 Department of Family Medicine, School of Medicine, University of Colorado Anschutz Medical Campus, Fitzsimons Building, 13001 East 17th Place, Aurora, CO 80045 USA

**Keywords:** Home environment, Parent, Child dietary intake, Fruit and vegetables, Sugar-sweetened beverages

## Abstract

**Background:**

Home environments shape children’s dietary habits, but which factors are most influential is unclear. The study purpose was to identify factors in the home environment associated with child intake of fruit and vegetables (FV) and sugar-sweetened beverages (SSBs) using a national dataset collected in 2013–2015 in the U.S.

**Methods:**

Data from 5,138 school-aged children (4–15 years old) from 130 U.S. communities were collected in 2013–2015. Parents and/or children completed a dietary screener and additional survey questions to assess household socioeconomic status (SES), grocery shopping sources, home food availability, social support for healthy eating, eating out frequency, and other home eating and related behaviors. Other child characteristics included breastfeeding history, intake of school foods, and participation in other nutrition programs. Community variables included predominant race/ethnicity and SES. Classification and regression trees (CART) identified key predictors of intake.

**Results:**

The FV and SSB CARTS had 14 and 12 terminal groups, respectively. Children with the highest FV intake (0.54 SD from mean cups/day; 13% of sample) had fruit more often available at home, dark green vegetables more often available at home, ate dinner with family more often, had SSBs less often available at home, and were breastfed longer. Conversely, children in the two groups with the lowest FV intake either had fruit less often available at home, and family never complimented their eating (-0.86; 2%), or they had family that rarely or sometimes complimented their eating, and perceived school lunches as unhealthy (-0.87; 1%). For SSB intake, the lowest consumers (-0.63 SD from mean tsp/day sugar; 17%) never or rarely had SSBs available at home, and lived in higher SES communities. Children in the two groups with the highest SSB intakes had SSBs available at home more often, and lived in a SNAP-participating household and either ate out less often, used a phone/computer for social networking, and had SSBs available at home very often (1.3; 1%), or they ate out more often, and were breastfed for a shorter duration (1.1; 5%).

**Conclusions:**

Home availability of FV and SSBs were the most salient predictors of intake of both FV and SSBs, while other predictors differed between FV and SSB intake. Study findings highlight several actionable home-environment strategies to test in future studies to improve school-aged children’s diets.

**Supplementary Information:**

The online version contains supplementary material available at 10.1186/s40795-026-01339-8.

## Background

Diets of children in the United States need to be improved to optimize health. Diet quality scores based on the Healthy Eating Index-2020 averaged 54 for children 5–8 years old and 51 for children 9–13 years old [1]. These scores are substantially below the maximum of 100 indicative of complete alignment with recommendations in the *Dietary Guidelines for Americans* [2]*.*

The home environment accounts for a substantial portion of child dietary intake, with an average of two-thirds of daily energy consumed at home [3]*.* While school and community environments also play a role, the home environment can have a stronger influence on establishing child dietary habits and preferences [4]*.* These influences are multifaceted, involving parenting practices, mealtime routines, and media use [5]*.* Family meals, for example, provide a significant opportunity to expose children to healthy foods, establish positive eating routines, and model and encourage healthy eating by parents and other household members [6]*.* Eating meals as a family have been associated with increased consumption of healthy foods, lower consumption of unhealthy foods high in added sugars and fat, and better overall diet quality of children [7]*.* The benefits of family meals can be reduced, however, by watching television (TV) while eating [8]*.* Exposure to food advertising can shape child food-related knowledge, preferences, and practices, leading to increased intake of snack foods and overall calories and decreased consumption of healthy foods [5]*.*

The home food environment, including the availability and accessibility of different types of foods and beverages, also influences child intake [5]*.* Children in homes where more fruit and vegetables (FV) are available are more likely to consume them [9]*.* Conversely, a home environment with readily available energy-dense, nutrient-poor foods, such as sugar-sweetened beverages (SSBs), can contribute to excessive energy intake [7]*.*

Socioeconomic status (SES) is an additional and broader contextual factor that can influence child diets [4]*.* Children from lower socioeconomic backgrounds are more likely to consume snacks, SSBs, and meals while watching TV than higher income peers [8]*.* Similarly, children living in food insecure households tend to have lower quality diets [10–13].

Effective and efficient interventions at the proximal family level can be improved by understanding which of these many home influences are the most salient predictors of child intake. Similarly, broader program and policy efforts to improve child nutrition may be improved by understanding family influences. Therefore, the purpose of this study was to use a national dataset, the Healthy Communities Study (HCS), to identify the home-related factors that predict child intake of FV and of SSBs, using an analytical technique designed to consider multiple factors simultaneously. These two dietary measures were selected because research consistently demonstrates that higher FV intake is associated with better health outcomes among children [14, 15], while higher consumption of SSBs correlates with adverse health [16]*.* While much has been learned from the HCS about the relationship between community and school programs and policies with child diets [17], the HCS data collected on the home environment have not previously been analyzed comprehensively.

## Methods

### Study design

Informed by the socioecological model to identify gaps and provide guidance for child obesity prevention efforts [18], the HCS was designed to identify characteristics and combinations of community programs and policies targeting childhood obesity as well as school, family, and home contextual factors related to BMI, diet, and physical activity among school-age children. The observational study was conducted between 2013 and 2015 across a diverse sample of communities in the U.S., as previously described [19, 20]. A hybrid sampling method was used to select 130 communities, defined as public high school catchment areas. This method combined a national probability-based sample of communities (n = 102) with a purposeful sample of "certainty" communities (n = 28). The probability-based sample was stratified based on race, ethnicity, income, region, and perceived program and policy intensity to ensure diversity. The "certainty" communities were identified by an expert panel as having evidence of innovative or promising programs and policies related to childhood obesity. This involved nomination, scoring, and selection by experts in obesity prevention. Within each study community, up to 2 elementary and 2 middle schools and up to 81 families were recruited to participate.

### Sampling of study participants (children and families)

A total sample of 5,138 elementary and middle school children (kindergarten through eighth grade, 4–15 years old) and their families participated in the study. Recruitment involved informational letters sent home and follow-up telephone calls. A stratified random selection of recruited children was conducted to maintain balance by sex, grade, and race/ethnicity, selecting one child per household. Children who were institutionalized, non-ambulatory, or whose families had lived in the community for less than 1 year were excluded.

### Data collection

Home visits were conducted by trained data collectors who administered a survey to parents and children (primary respondents were parent/adult proxy with child assistance for children 4–8 years old; children 9–11 years old, with assistance from the parent/proxy; and children 12 and older, with input from parent/proxy if needed).

Outcome variables: A 27-item version of the National Health and Nutrition Examination Survey (NHANES) Dietary Screener Questionnaire was administered to assess usual dietary intake, including our two outcomes of interest, FV and SSB intake [21, 22]. Respondents reported the frequency of consuming select foods and beverages over the past month (30 days). Quantities consumed were estimated from reported frequencies using scoring algorithms developed by the National Cancer Institute, based on sex and age specific intakes from 2010 NHANES data [23]. Daily cup equivalents of FV were derived from 9 items including legumes, but excluding fried potatoes: fruit; fruit juice; green leafy salad; other potatoes; beans; other vegetables; salsa; tomato sauces; and vegetables/tomato sauce from pizza. Daily tsp of sugar from SSB were derived from 4 items: soda/soft drinks; sweetened coffee/tea; sports/energy drinks; and fruit drinks (flavored milk was not included).

Predictor variables (Table 1): Predictor variables were identified based on the socioecological model which recognizes that the home environment interacts with broader aspects of family circumstances to influence child dietary intake [24]. Candidate variables were selected based on reviews of the literature by the study team and prioritized in consultation with an HCS nutrition expert subcommittee of over a dozen nutrition and dietary assessment experts, as well as an HCS observational study monitoring board, as previously described [19, 25]. Survey questions from pre-existing instruments from published sources were used whenever possible [25, 26]. Questions asked about household socioeconomic status including participation in the Supplemental Nutrition Assistance Program (SNAP) and Special Supplemental Nutrition Program for Women, Infants and Children (WIC), and household food insecurity [27, 28], sources where primary shopper usually buys groceries for the family, perceptions about produce availability [29], the availability of select foods in the home [30], social support for healthy eating by family and friends [31], eating out frequency [29], home eating behaviors (e.g., frequency of breakfast eating, family dinners, eating fast food, eating while watching TV) [31, 32], and other behaviors which may influence eating through exposure to food stores and/or food marketing) [25]. Other questions about factors which may relate to child dietary intake included: breastfeeding history [30], school food exposure and perceptions of healthiness [33, 34], and child participation in programs to support healthy eating (questions developed for the HCS).

**Table 1:** Independent variables in the home and larger environment included in the classification and regression tree analyses on child dietary intake (*n* = 5,035–5,077 children)

VARIABLE	DESCRIPTION	MEAN (SD)	Number^1^	Percent
HOME ENVIRONMENT				
Household socioeconomic status				
Supplemental Nutrition Assistance Program (SNAP) participation (past month)	0 = no; 1 = yes	0.39 (0.49)	3073 1951	61.2 38.8
Special Supplemental Nutrition Program for Women, Infants and Children (WIC) participation (past month)	0 = no; 1 = yes	0.19 (0.39)	4060 961	80.9 19.1
Household food insecurity (past 12 months)	Range 0–6 (responses to two questions, worry food would run out, food did not last, summed each scored as 0 = often true, 1 = sometimes true, 3 = never true)	5.06 (1.21)		
Grocery shopping sources				
Large chain grocery store or supermarket	1 = never;	4.20 (0.92)	101	2.0
	2 = rarely;		137	2.7
	3 = sometimes;		698	13.8
	4 = often;		1840	36.4
	5 = very often		2285	45.1
Natural or organic supermarket	1 = never;	1.98 (1.17)	2457	48.6
	2 = rarely;		1080	21.3
	3 = sometimes;		888	17.5
	4 = often;		417	8.2
	5 = very often		218	4.3
Convenience store^2^	0 = very often;	3.17 (1.00)	101	2.0
	1 = often;		261	5.2
	2 = sometimes;		803	15.9
	3 = rarely;		1426	28.2
	4 = never		2468	48.8
Small local or corner store	1 = never;	2.46 (1.20)	1415	28.0
	2 = rarely;		1225	24.2
	3 = sometimes;		1409	27.8
	4 = often;		705	13.9
	5 = very often		306	6.0
Warehouse club store	1 = never;	2.60 (1.31)	1501	29.7
	2 = rarely;		812	16.1
	3 = sometimes;		1420	28.1
	4 = often;		864	17.1
	5 = very often		462	9.1
Discount superstore	1 = never;	3.45 (1.16)	374	7.4
	2 = rarely;		603	11.9
	3 = sometimes;		1511	29.9
	4 = often;		1494	29.5
	5 = very often		1074	21.2
Online delivery	1 = never;	1.12 (0.49)	4685	92.6
	2 = rarely;		217	4.3
	3 = sometimes;		100	2.0
	4 = often;		38	0.8
	5 = very often		19	0.4
Ethnic market	1 = never;	1.72 (1.13)	3286	65.0
	2 = rarely;		623	12.3
	3 = sometimes;		627	12.4
	4 = often;		341	6.7
	5 = very often		180	3.6
Farmers market/co-op	1 = never;	2.02 (1.09)	2239	44.3
	2 = rarely;		1080	21.4
	3 = sometimes;		1266	25.0
	4 = often;		347	6.9
	5 = very often		126	2.5
Ease to buy, large selection and high quality of fresh fruit and vegetables available in community	Range: 2–15 (responses to three questions summed with each scored as 1 = disagree a lot, 2 = disagree a little, 4 = agree a little; 5 = agree a lot)	12.70 (3.21)		
Home food availability				
Fruit	1 = never;	4.52 (0.71)	7	0.1
	2 = rarely;		49	1.0
	3 = sometimes;		462	9.1
	4 = often;		1323	26.1
	5 = very often		3224	63.7
Dark green vegetables	1 = never;	4.07 (0.94)	53	1.0
	2 = rarely;		239	4.7
	3 = sometimes;		1056	20.8
	4 = often;		1694	33.4
	5 = very often		2024	40.0
1% or fat-free milk	1 = never;	2.83 (1.75)	2047	40.4
	2 = rarely;		514	10.1
	3 = sometimes;		402	7.9
	4 = often;		483	9.5
	5 = very often		1621	32.0
Salty snacks^2^	0 = very often;	1.63 (1.07)	908	17.9
	1 = often;		1281	25.3
	2 = sometimes;		1847	36.5
	3 = rarely;		851	16.8
	4 = never		178	3.5
Sugar-sweetened beverages^2^	0 = very often;	1.99 (1.23)	723	14.3
	1 = often;		1040	20.5
	2 = sometimes;		1478	29.2
	3 = rarely;		1195	23.6
	4 = never		630	12.4
Social support for healthy eating				
Family compliments child’s eating habits	1 = never;	2.94 (1.29)	979	19.3
	2 = rarely;		759	15.0
	3 = sometimes;		1657	32.7
	4 = often;		967	19.1
	5 = very often		712	14.0
Friends compliment child’s eating habits^3^	1 = never;	1.75 (1.06)	763	59.5
	2 = rarely;		216	16.8
	3 = sometimes;		197	15.4
	4 = often;		81	6.3
	5 = very often		26	2.0
Family encourages child to eat fruit and vegetables when tempted not to	1 = never;	3.67 (1.24)	474	9.3
	2 = rarely;		334	6.6
	3 = sometimes;		1168	23.0
	4 = often;		1515	29.8
	5 = very often		1590	31.3
Friends encourage child to eat fruit and vegetables when tempted not to^3^	1 = never;	1.76 (1.08)	758	58.9
	2 = rarely;		224	17.4
	3 = sometimes;		190	14.8
	4 = often;		78	6.1
	5 = very often		36	2.8
Eating out frequency				
Frequency household eats out at 7 types of eateries (full service restaurant, buffet/cafeteria, fast food restaurant, deli, convenience store, bar/tavern/lounge, coffee shop)	Range: 1–5 (responses to each of 7 types of eateries averaged with each scored as 1 = very often; 2 = often; 3 = sometimes; 4 = rarely; 5 = never)	2.04 (0.47)		
Frequency child eats or drinks from a fast food restaurant	Range: 0–7 (scored as 0 = 7 days, 1 = 6 days; 2 = 5 days; 3 = 4 days; 5 = 3 days; 6 = 2 days; 7 = 1 day in past week)	5.98 (1.18)		
Home eating behaviors				
Frequency child eats breakfast	Range: 0–7 days in past week	6.14 (1.76)		
Frequency family eats dinner together	Range: 0–7 days in past week	5.02 (2.43)		
Frequency TV on during meals^2^	1 = never;	3.40 (1.40)	941	18.5
	2 = rarely;		816	16.0
	3 = sometimes;		1344	26.4
	4 = often;		838	16.4
	5 = very often		1157	22.7
Other behaviors (that may influence eating through exposure to food stores and/or food marketing)				
Frequency child walks/bikes to/from school	Range: 0–5 days in past week	0.90 (1.79)		
Frequency child walks/bikes to/from store, park, playground or friend’s house	Range: 0–7 days in past week	1.45 (2.11)		
Frequency child uses computer for games/playing on internet (not for schoolwork or social networking)	Range: 0–7 days in past week	1.53 (2.61)		
Frequency child uses computer or phone for social networking	Range: 0–7 days in past week	3.19 (2.25)		
Frequency child watches TV	Range: 0–7 days in past week	5.30 (2.33)		
LARGER ENVIROMENT				
Child breastfeeding history				
Ever breastfed or fed breastmilk	0 = no; 1 = yes	0.73 (0.44)	1355 3672	27.0 73.0
Age stopped breastmilk	Range: 0–8 years	0.59 (0.70)		
School foods				
Frequency eating school meals (breakfast and lunch)	Range: 0–10 meals in past week	5.98 (3.56)		
School lunch healthy	1 = never;	3.27 (1.15)	424	8.7
	2 = rarely;		627	12.8
	3 = sometimes;		1899	38.8
	4 = often;		1078	22.0
	5 = very often		865	17.7
Other foods (not part of school meal programs) sold at school healthy	1 = never;	2.53 (1.31)	697	29.8
	2 = rarely;		487	20.8
	3 = sometimes;		612	26.2
	4 = often;		305	13.1
	5 = very often		236	10.1
Child participation in programs that encourage healthy eating				
Frequency of participation (past 6 months)	1 = never;	2.46 (1.44)	2089	41.0
	2 = rarely;		514	10.1
	3 = sometimes;		1124	22.1
	4 = often;		760	14.9
	5 = very often		602	11.8
Number of programs in community (past 6 months)	Range: 1–5	1.77 (1.04)		
Community characteristics				
Race/ethnicity	0 = predominantly White (both Hispanic and African American < 46%);	0.83 (0.90)	2600	50.6
	1 = predominantly Black (African American ≥ 46%, Hispanic < 46%);		800	15.6
	2 = predominantly Hispanic (Hispanic ≥ 46%, African American < 46%)		1738	33.8
Community socioeconomic status^4^	1 = high;	1.97 (0.82)	1801	35.1
	2 = medium;		1671	32.5
	3 = low		1666	32.4

^1^The sum of the frequencies varies and the range may deviate from what is theoretically possible, due to differences in the number of missing values for each variable

^2^Reverse coded from other similar variables

^3^Asked only of children 12–15 years old

^4^Community was defined by high school catchment area; Six variables (i.e., percent of population with poverty, percent of population with education of high school or higher, percent of population in labor force 16 years and over who were unemployed, percent of population in renter-occupied housing units, percent of vacant housing units, and percent of population with health insurance) were combined using a factor analysis with one factor. The resulting factor regression score was divided into terciles to create three categories of socioeconomic status

Community race/ethnicity and SES were also factored into the analysis based on previous HCS analyses showing that these community indicators were modifiers of associations between a composite score of attributes of community programs and policies with longitudinal changes in body mass index [35]. Using data from the 2009–2013 five-year American Community Survey, community race/ethnicity and SES were area-weighted according to the proportion of each census tract located within the community’s catchment zone, following the methodology outlined by Strauss et al. [20]. Communities were grouped into three racial/ethnic categories: predominantly African American (where at least 46% of residents were African American and fewer than 46% were Hispanic); predominantly Hispanic (those with at least 46% Hispanic residents and fewer than 46% African American); and predominantly non-Hispanic White, hereafter “White” (those with fewer than 46% Hispanic and fewer than 46% non-Hispanic African American residents). Socioeconomic status was divided into low, medium, and high categories, determined by six variables combined using a one-factor analytic model: the percentage of individuals living below the poverty line, the percentage who completed high school or higher education, unemployment rates among those 16 and older in the labor force, the proportion of renters, housing vacancy rates, and the percentage of the population with health insurance (public or private).

### Data analysis

Classification and Regression Tree (CART) analyses were performed separately for child FV and SSB intakes. CART is a versatile, nonparametric machine-learning technique that addresses many of the challenges faced by traditional regression methods, especially their limitations in dealing with complex variable interactions [36]. To control for known demographic correlates of FV and SSB intake and ensure splits reflect other predictors, the analysis began with fitting regression models for each outcome, accounting for child age and sex. Standardized residuals from these models were then used as the outcome variables in the CART models. At each stage, the CART algorithm selected the predictor and cutoff value that resulted in the largest reduction in mean squared prediction error [37]. Splitting continued until the stopping criterion was met, defined using cost-complexity pruning with the optimal complexity parameter chosen via 50-fold cross-validation [38].

## Results

Of the 5,138 school-aged children in the HCS dataset, 5,035 (98.0%) had complete data to examine home characteristics in relation to child FV intake, and 5,077 (98.8%) had complete data to examine child SSB intake. The mean age of the children in the total sample was 9.3 years. The proportion of boys was 48.2%. In terms of ethnicity, 53.9% were Hispanic. The racial composition was 56.9% White, 18.7% African American, 9% other (including more than one race), and 15.4% did not respond. Mean intakes (SD) were 2.5 (0.9) cup equivalents/day from FV and 7.0 (4.8) tsp sugar/day from SSBs.

### FV intake

**Fig. 1:**
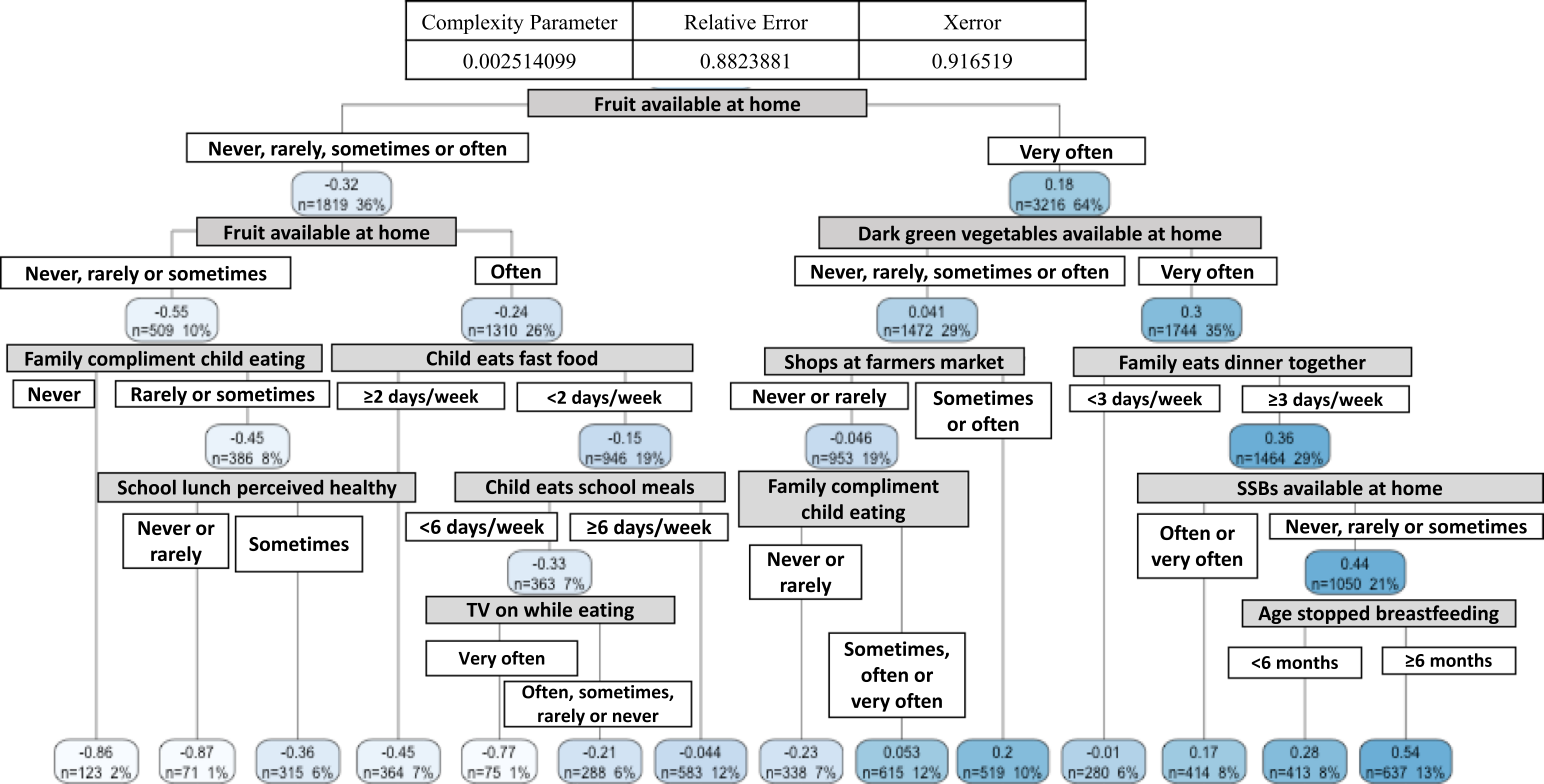
Classification and Regression Tree (CART) predicting child intake (cups/day) of fruit and vegetables (model with 13 splits, *n* = 5,035 children). Footnotes: Predictors are in grey boxes followed by their values in white boxes. Values in rounded boxes are intake (standard deviations) with darker blue signifying higher intake relative to mean, followed by sample size and percent of total sample. Relative Error, proportion of unexplained error (model based). Xerror, proportion of unexplained error (based on 1000 fold cross-validation). Complexity parameter, a tuning value in CART that balances tree size against prediction accuracy. Abbreviations: SSBs, sugar-sweetened beverages; TV, television

The CART for FV had 13 splits with 14 terminal groups of children. The group average of the standardized residuals (or standard deviation (SD)) for FV intake ranged from −0.87 to 0.54 cups per day, and the group size ranged from 1 to 13% of the study sample (Fig. 1). Fruit availability at home was the first splitting variable: children with fruit available at home very often had a higher FV intake (0.18, 64% of children) compared to those who less frequently had fruit available at home (−0.32, 36%).

Focusing first on the terminal groups on the left side of the CART where FV intakes were lower, if fruit was available at home sometimes or less, and the family never complimented the child’s eating habits, then FV intake was −0.86 (2% of children). If fruit was available at home sometimes or less, but the family complimented the child’s eating habits at least rarely, and the school lunch was perceived as never or rarely healthy, then FV intake was −0.87 (1%). If the statement above were true, but the school lunch was perceived as healthy at least sometimes, then FV intake was −0.36 (6%).

If fruit was available at home often, and the child ate fast food 2 or more days/week, then FV intake was −0.45 (7% of children). If the statement above were true and the child ate school meals less than 6 days/week, and ate at home while watching TV very often, then FV intake was −0.77 (1% of children). However, if the child ate at home while watching TV often, sometimes, rarely or never, then FV intake was −0.21 (6%). If fruit was available at home often, the child ate fast food less than 2 days/week, and ate school meals 6 or more days/week, then FV intake was −0.044 (12%).

Moving to the right side of the CART where FV intakes were higher, if fruit was available at home very often, dark green vegetables were available at home often or less, the primary food shopper in the household shopped at a farmers market/co-op never or rarely, and the family complimented the child’s eating habits never or rarely, then FV intake was −0.23 (7% of children). If the statement above were true, but the family complimented the child’s eating habits at least sometimes, then FV intake was 0.058 (12%). If fruit was available at home very often, dark green vegetables were available at home often or less, and at least sometimes the primary food shopper shopped at a farmers market/co-op, then FV intake was 0.2 (10%).

If both fruit and dark green vegetables were available at home very often, and the family ate dinner together less than 3 days/week, then FV intake was −0.01 (6% of children). If both fruit and dark green vegetables were available at home very often, the family ate dinner together more than 3 days/week, and SSBs were available at home often or very often, then FV intake was 0.17 (8%). If the statement above were true, but SSBs were available at home sometimes at most, and the child stopped breastfeeding before 6 months of age, then FV intake was 0.28 (8%). However, if the child was breastfed for at least 6 months, then FV intake was 0.54 (13%).

### SSB intake

**Fig. 2:**
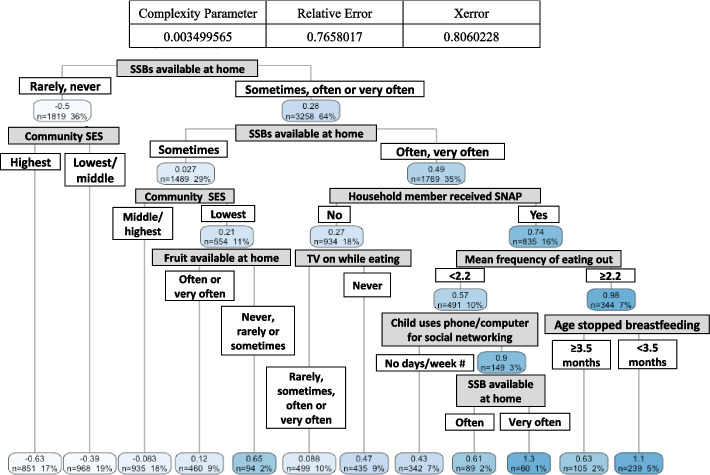
Classification and Regression Tree (CART) predicting child intake (tsp sugar/day) of sugar-sweetened beverages (model with 11 splits, *n* = 5,077 children). Footnotes: Predictors are in grey boxes followed by their values in white boxes. Values in the rounded boxes are intake (standard deviations) with darker blue signifying higher intake relative to mean, followed by sample size and percent of total sample. Relative Error, proportion of unexplained error (model based). Xerror, proportion of unexplained error (based on 1000 fold cross-validation). Complexity parameter, a tuning value in CART that balances tree size against prediction accuracy. Abbreviations: SES, socioeconomic status; SSBs, sugar-sweetened beverages. Frequency of eating out determined from responses to each of 7 types of eateries summed with each scored as 1=very often; 2=often; 3=sometimes; 4=rarely; 5=never. #Alternate value ( ≥1 day/week) not shown

The CART for SSB intake had 11 splits with 12 terminal groups of children. The group average SD for SSB intake ranged from −0.63 to 1.3 tsp/day of sugar, and the group size ranged from 1 to 19% of the study sample (Fig. 2). SSB availability at home was the first splitting variable: children with SSBs available at home rarely or never had a lower SSB intake (−0.5, 36% of children) compared to those with SSBs available at home sometimes, often or very often (0.28, 64%).

Focusing on the terminal groups on the left side of the CART where SSB intakes were lower, if SSBs were available at home rarely or never, and children were in a community in the highest SES category, then SSB intake was −0.63 (17% of children). If SSBs were available at home rarely or never, and children were in a community in the lowest or middle SES categories, then SSB intake was −0.39 (19%).

Moving to the right on the CART, if SSBs were available at home sometimes, and children were in a community in the middle or highest SES categories, then SSB intake was −0.083 (18% of children). If the statement above were true, but the child lived in a community in the lowest SES category, and fruit was available at home often or very often, then SSB intake was 0.12 (9%). However, if fruit was available at home never, rarely or sometimes, then SSB intake was 0.65 (2%).

Continuing further to the right, if SSBs were available at home often or very often, no one in the household received SNAP, and the child ate while watching TV rarely, sometimes, often or very often, then SSB intake was 0.088 (10% of children). If the statement above were true, but the child never ate while watching TV at home, then SSB intake 0.47 (9%).

If SSBs were available at home often or very often, someone in the household received SNAP, the household ate out less frequently, and the child did not use a computer or phone for social networking in the past week, then SSB intake was 0.43 (7% of children). If the statement above were true, and the child used a computer or phone for social networking on at least 1 day in the past week, and SSBs were available at home often, then SSB intake was 0.61 (2%). However, if SSBs were available at home very often, then SSB intake was 1.3 (1%).

Finally, if SSBs were available at home often or very often, someone in the household received SNAP, the household ate out more frequently, and the child stopped breastfeeding at 3.5 months of age or later, then SSB intake was 0.63 (2% of children). If the statement above were true, but the child stopped breastfeeding before 3.5 months of age, then SSB intake was 1.1 (5%).

## Discussion

This study used a national dataset to examine multiple home environmental factors in relation to school-aged child dietary intakes of FV and SSBs. Unlike standard modeling methods, CART analysis does not require pre-selection of variables or adjustment for multiple comparisons; instead, it evaluates all available predictors and automatically selects those that contribute most to the outcome. Therefore, we examined all potentially relevant home environmental measures collected in the HCS including household SES, grocery shopping sources, home food availability, social support for healthy eating, eating out, and other family habits. Based on prior literature showing relationships with child dietary intake, we also included measures of breastfeeding [39, 40], school meals [12], and participation in programs that encourage healthy eating [41], as well as predominant community race/ethnicity and SES [42, 43].

Among the 41 measures considered, home availability of FV and SSBs were the most salient (first-selected) variables related to intakes of school-aged children – with more frequent home availability of fruit and dark green vegetables associated with higher FV intakes, and more frequent home availability of SSBs associated with higher SSB intakes. Moreover, albeit lower in the models, less home availability of fruit was related to lower SSB intake, and more home availability of SSBs was related to lower FV intake. Using a different analytical method, our study extended the conclusion of systematic reviews [44–47] – that home food availability is a critical determinant of child intake. While the HCS did not measure the dietary habits of parents, home food availability may reflect parent dietary preferences and modeling, which have been shown to predict child intake [44, 45]. Of note, home availability of 1% or fat-free milk and salty snacks were not related to FV and SSB intakes. Future studies are needed to better understand the influential role of home availability of different types of foods and beverages on children. Studies across all age groups, including those younger and older than the 4–13 year olds in the HCS, are also warranted given that the home environment can have differential impacts as children get older and gain more autonomy in food access and selection [48]. In addition, because food purchasing and consumption are proximal behaviors, improving home food availability alone may be insufficient to meaningfully change intake unless individual factors (e.g., self-efficacy for preparing FV, perceived consequences of consuming SSBs) are also addressed.

Community SES and household SNAP participation were other high-salient variables related to SSB intake, but not to FV intake. SSBs, a convenient and relatively inexpensive source of dietary energy [49], have been shown to be more readily available and more heavily marketed to lower income populations in the United States [50–52]. The influence of marketing is also suggested by the relation of eating out and social networking online being lower-salient factors related to child SSB intake, but not to FV intake in the present study. While FV quality has been shown to differ by community SES, FV availability and price have not been as consistently related [53–55]. More frequently having the TV on while eating at home was related unexpectedly to lower intakes of both SSBs and FV. Other studies have shown that the habit of eating while watching TV and exposure to food advertising, most of which is for unhealthy foods [56], is related to higher SSB intakes [8, 57, 58]. The reason for our unexpected finding is unclear, but more frequent use by children of a phone or computer for social networking was associated with higher SSB intake in the present study.

Several measures were related to higher FV intake, but did not appear in the SSB CART. Higher child FV intakes were related to more frequent shopping at a farmers market, family complimenting child eating habits, dinner with family, eating school meals, and perceiving school meals to be healthy. Indeed, shopping at farmers markets was the only grocery shopping source predictive of child FV intake and no grocery shopping sources appeared in the SSB CART. Shopping at farmers markets, which typically offer a high proportion of products as FV, has been related to increased FV intake [59]. Since greater access to farmers markets has been documented in higher income communities in the U.S. [60], more frequent shopping at farmers markets may reflect other health-promoting characteristics associated with higher incomes. While convenience stores have been shown to sell a less healthy range of products compared to other grocery outlets [61], use of convenience stores was relatively low in the HCS sample in comparison to large chain grocery stores/supermarkets and discount superstores. School meals (perceptions or healthfulness, and frequency of consumption) appeared in the FV CART and not the SSB CART, an expected finding because reimbursable school meals in the U.S. must provide specified amounts of FV and cannot serve SSBs other than flavored milk [62]. Indeed, school meals have been shown to be the highest nutritional quality of children’s food sources [63]. Family complimenting of child’s eating habits was associated with higher FV intake while the related constructs, friends complimenting child’s eating habits and family and family or friends encouraging the child to eat FV, were not. More research is needed to understand the influence of different types of social support for healthy eating.

A longer breastfeeding duration was a lower-salient variable related to both higher FV and lower SSB intakes. Other studies have shown breastfeeding related to young children’s diet quality, but this relationship tends to diminish as children get older [39, 40]. An association between breastfeeding and subsequent child diet may reflect a lasting effect of more exposure to diverse flavors in breastmilk [64], and may be an indicator of a more health conscious household [65]. Similarly, eating out more frequently at a range of restaurants by the family and specifically at a fast food restaurant by the child, were related to a higher intake of SSBs and a lower intake of FV, respectively. Others have similarly found associations between eating out and poorer diet quality in children using different samples and study designs [66–68].

Strengths of this study include a large, diverse sample of school-aged children from multiple communities across the U.S., with representation of communities with relatively high proportions of Hispanic, African American and lower income populations, and use of an analytical method that can discriminate among the multiple home and select school and community factors measured. The cross-sectional observational design, however, means that we cannot make causal inferences. Dietary intake, although quantified for the prior month, was measured at only a single point in time for each study child, and both outcome and most predictor variables were self-reported by parents and/or children and are therefore subject to recall and social desirability bias. The predictive factors may have differed between fruit and vegetables; however, the dietary assessment method used did not allow for separate examination of fruit from vegetables. Other home-related factors that can influence child diets, such as parent modeling and knowledge of healthy eating, family functioning, and parental control of child feeding, were not assessed in the HCS due to constraints to the survey length. The CART procedure considered all the variables available and allowed for complex interactions among these variables, and the cross-validation algorithm ensured that results were robust given the variables available. The inclusion of some important variables either not available or available but poorly measured could have altered the results. HCS data were collected over 10 years ago. While relatively small changes in children’s overall diet quality have been observed nationally since 2013 [69], it is possible that other components of the home environment would be predictive of children’s intake today. Future studies are needed once more current U.S. datasets with comparable home measures are available.

## Conclusions

More frequent shopping at farmers markets, eating dinner together as a family, family complimenting child eating, and promotion of eating healthy school meals appear to be predictors of higher child FV intake. More home availability of FV, less home availability of SSBs, as well as less frequently eating out or eating at a fast food restaurant and longer breastfeeding duration predicted lower SSB intake and higher FV intake. Family-based interventions are needed to test if these are actionable strategies to improve the diets of school-aged children.

## Supplementary Information

Additional file 1.

## Data Availability

The dataset analyzed during the current study and study protocol and forms are available in the National Heart, Lung, and Blood Institute repository. [https://biolincc.nhlbi.nih.gov/studies/hcs/].

## Abbreviations

CART: Classification and regression tree
FV: Fruit and vegetables
HCS: Healthy Communities Study
NHANES: National Health and Nutrition Examination Survey
SES: Socioeconomic status
SNAP: Supplemental Nutrition Assistance Program
SSBs: Sugar-sweetened beverages
TV: Television
